# Parallelized multi‐stimuli bioreactor for combined, physiologically relevant fluid shear stress and intermittent hydrostatic pressure in 3D bone tissue models

**DOI:** 10.1002/btm2.70184

**Published:** 2026-10-28

**Authors:** Stefano Gabetti, Farah Daou, Beatrice Masante, Ranveer Kaur, Simone Israel, Shahd Alahmad, Elisa Scatena, Carlotta Achille, Raffaella Pecci, Costantino Del Gaudio Gaudio, Martina Birocco, Andrea Cochis, Lia Rimondini, Cristina Bignardi, Diana Massai

**Affiliations:** ^1^ Department of Mechanical and Aerospace Engineering and PolitoBIOMed Lab Politecnico di Torino Turin Italy; ^2^ Centro 3R, Interuniversity Center for the Promotion of the 3Rs Principles in Teaching and Research Turin Italy; ^3^ Department of Health Sciences Center for Translational Research on Autoimmune and Allergic Diseases (CAAD), Università del Piemonte Orientale (UPO) Novara Italy; ^4^ E. Amaldi Foundation Rome Italy; ^5^ Hypatia Research Consortium Rome Italy; ^6^ National Center for Artificial Intelligence and Innovative Technologies for Health, Istituto Superiore di Sanità Rome Italy; ^7^ Italian Space Agency Rome Italy; ^8^ Laboratory for High Performance Ceramics, Empa—Swiss Federal Laboratories for Materials Science and Technology Dübendorf Switzerland

**Keywords:** 3D bone tissue models, bone tissue engineering, fluid flow‐induced shear stress, intermittent pressure, mechanotransduction, multi‐stimuli bioreactor

## Abstract

Regeneration of bone tissue is tightly regulated by its complex mechanical microenvironment, in which intraosseous hydrostatic pressure (HP) and interstitial fluid flow‐induced shear stress (FSS) represent key mechanical cues. While the individual effects of these stimuli have been extensively investigated, their combined influence within physiologically relevant three‐dimensional (3D) bone tissue models remains largely unexplored due to the lack of suitable experimental platforms. In this study, we present an automated, parallelized multi‐stimuli bioreactor designed to deliver tunable combinations of FSS and intermittent HP to 3D bone tissue models. The platform integrates a direct perfusion bioreactor with a custom‐designed, modular intermittent HP unit based on a solenoid pinch valve, enabling independent and combined control of flow rate and pressure waveforms. Bioreactor performance was characterized using two biomimetic polylactic acid (PLA) scaffold types, demonstrating the ability of the system to reproduce physiologically relevant ranges of HP (1.81–30.59 kPa) and FSS (0.48–683.26 mPa). Biological experiments using bone marrow‐derived mesenchymal stem cells (BM‐MSCs) suspended in a collagen matrix and seeded into PLA scaffolds, cultured for 7 days, showed that dynamic culture conditions significantly enhanced cell viability compared with static culture. Moreover, the combination of FSS and intermittent HP stimulation within daily activity‐related ranges accelerated early osteogenic differentiation compared to direct perfusion alone. Overall, the proposed platform provides a versatile and robust investigation tool for advancing bone mechanobiology and tissue engineering research.

**Translational Impact Statement**

This work introduces an automated, parallelized multi‐stimuli bioreactor that reproduces key aspects of the human bone mechanical environment by delivering tunable and combinable fluid shear stress and hydrostatic pressure to three‐dimensional bone tissue models. We demonstrated that the combined application of these mechanical cues promoted early osteogenic differentiation more effectively than perfusion alone. This research platform supports the development and testing of bone regeneration strategies, biomaterials, and therapies, enabling more reliable translation from laboratory models to clinical and orthopedic applications.

## 1 INTRODUCTION

Routine activities expose the human skeletal system to varying mechanical loads, resulting in bone tissue deformations ranging from approximately 0.04% during daily activities to about 0.2% during active exercise.^1^, ^2^ These strains generate regions of compression and tension, leading to variations in intraosseous hydrostatic pressure (HP) distribution^3^, ^4^ that can be directly sensed by bone cells. Moreover, pressure gradients drive interstitial fluid flow within the lacuno‐canalicular network, thereby generating fluid flow‐induced shear stress (FSS) on bone cells.^5^, ^6^ Thus, at the microscale, bone cells are exposed to a combination of intermittent HP (~3–8 kPa during daily activity and up to ~10–35 kPa during active exercise^4^, ^7^, ^8^, ^9^, ^10^) and FSS (~20–5000 mPa^11^, ^12^, ^13^, ^14^), both of which play a crucial role in maintaining bone mass and skeletal integrity and in regulating bone remodeling.^15^, ^16^, ^17^, ^18^

Several studies have investigated the influence of specific mechanical stimuli on bone biology,^19^, ^20^, ^21^, ^22^, ^23^, ^24^, ^25^ however much remains to be uncovered regarding how the overall mechanical microenvironment of bone tissue regulates stem cell behavior^26^ and which mechanotransduction mechanisms drive bone remodeling. Addressing these questions would be crucial for advancing bone repair strategies.

In this context, bone tissue engineering (BTE) represents a powerful approach for the development of functional in vitro bone tissue models, supporting both basic research and translational applications. From the cellular perspective, bone marrow‐derived mesenchymal stem cells (BM‐MSCs) play a central role in bone regeneration and are particularly promising for scaffold‐based bone tissue models, owing to their multipotency and ability to differentiate into osteoblasts.^27^ Considering tissue architecture, the use of three‐dimensional (3D) scaffolds recapitulating key bone features has consistently been shown to outperform traditional two‐dimensional (2D) approaches in the study of bone biology.^28^, ^29^ Indeed, the porosity, pore size, and interconnectivity of the scaffold not only determine the mechanical and permeability properties of bone‐like constructs but also critically influence cell behavior by modulating key processes, including vascularization, osteogenesis, and immune modulation.^30^, ^31^, ^32^, ^33^, ^34^

Ultimately, to reproduce in vitro the complex dynamic microenvironment of bone tissue, bioreactors designed for delivering specific mechanical stimuli in a controlled and physiologically relevant manner are required.^35^ To date, bioreactors providing intermittent HP have significantly advanced investigations in bone mechanobiology.^36^ Early pneumatic devices applying cyclic HP to 2D cell cultures allowed the observation of enhanced osteogenic marker expression and mineral deposition in osteoblastic cells,^37^, ^38^, ^39^ as well as early osteogenic commitment in BM‐MSCs.^40^ The subsequent development of pneumatic bioreactors capable of delivering intermittent HP to 3D in vitro bone models^41^, ^42^ enabled the investigation of combined mechanical and topographical cues. In particular, intermittent HP (peak pressure = 270 kPa, frequency = 1 Hz) was shown to promote MSCs' osteogenic differentiation on polycaprolactone (PCL) nanofibers^43^ and in collagen hydrogels.^44^

Beyond pressure‐only systems, further modular bioreactors have enabled the exploration of different stimuli on 2D cell cultures. Stavenschi et al. developed a flow chamber‐based bioreactor to apply either oscillatory shear regimes^45^ or cyclic HP^46^ to cells. Although the bioreactor could only deliver a single stimulus at a time, the results demonstrated that cyclic stimulation elicits a positive osteogenic response in MSCs, particularly at 2 Hz. Gardinier et al. advanced this approach by proposing a parallel plate flow chamber for delivering FSS while simultaneously applying varied magnitudes of HP on MC3T3‐E1 osteoblasts‐like cells. They demonstrated that HP during FSS can regulate purinergic signaling and actin cytoskeleton reorganization in osteoblasts in a biphasic manner.^47^ More recently, Zhou et al. developed a mechanical stimulation device for concurrently exposing cells to FSS and cyclic HP. These combined stimuli synergistically promoted the osteogenic differentiation of BM‐MSCs, with the activation of stimulus‐specific signaling pathways.^48^

Despite these advances, bioreactors capable of delivering combined mechanical stimuli remain largely restricted to 2D cell cultures. To the best of our knowledge, no existing device enables the application of FSS together with intermittent HP to 3D bone tissue models, leaving a critical gap in the ability to recapitulate in vitro the multifactorial mechanical environment of bone tissue. Comprehensive studies integrating scaffold‐based bone tissue models with dynamic, physiologically relevant mechanical loading remain notably scarce, despite being crucial for unraveling the mechanotransduction pathways involved in bone tissue regeneration.^49^

Inspired by this scenario, we propose an automated, parallelized multi‐stimuli bioreactor for delivering tunable combinations of FSS and intermittent HP to in vitro 3D bone tissue models. Specifically, building upon a direct perfusion bioreactor previously developed for delivering controlled FSS,^24^ we designed and integrated a custom intermittent HP unit for providing tunable HP stimulation. Once assembled, the FSS & intermittent HP bioreactor was characterized in terms of the achievable HP and FSS ranges as a function of two different biomimetic scaffold types. Finally, using this experimental platform, we investigated the effects of combined FSS and intermittent HP on the early biological response of biomimetic scaffolds seeded with BM‐MSCs, focusing on cell viability, cell distribution, and gene expression.

## 2 MATERIALS AND METHODS

### 2.1 FSS and intermittent HP bioreactor

To replicate the multi‐physical microenvironment of bone tissue, a direct perfusion bioreactor,^24^ was upgraded to deliver tunable combinations of physiologically relevant FSS and intermittent HP to 3D bone tissue models in an automated and parallelizable manner. Specifically, the bioreactor comprises three independent culture chambers (CCs), each incorporated into a dedicated closed‐loop hydraulic circuit. Each CC was manufactured by stereolithography (Dental SG Resin, Formlabs, USA) and consists of two screwable components designed to accommodate constructs of various sizes, which are secured in place by tailored polydimethylsiloxane (PDMS) holders (SYLGARD 184, Dow Corning, USA) and directly perfused from bottom to top (Figure 1a). Each hydraulic circuit is connected to a multichannel peristaltic pump (G100‐1J, Longer Precision Pump, China), which provides flow rates ranging from 0.024 to 24 mL/min to enable tunable FSS stimulation. Each circuit further includes oxygen‐permeable tubing (Darwin Microfluidics, France), a medium reservoir equipped with a disposable air filter (Sartorius, Germany), and a custom intermittent HP unit designed to deliver bone‐like HP stimulation.

**FIGURE 1:**
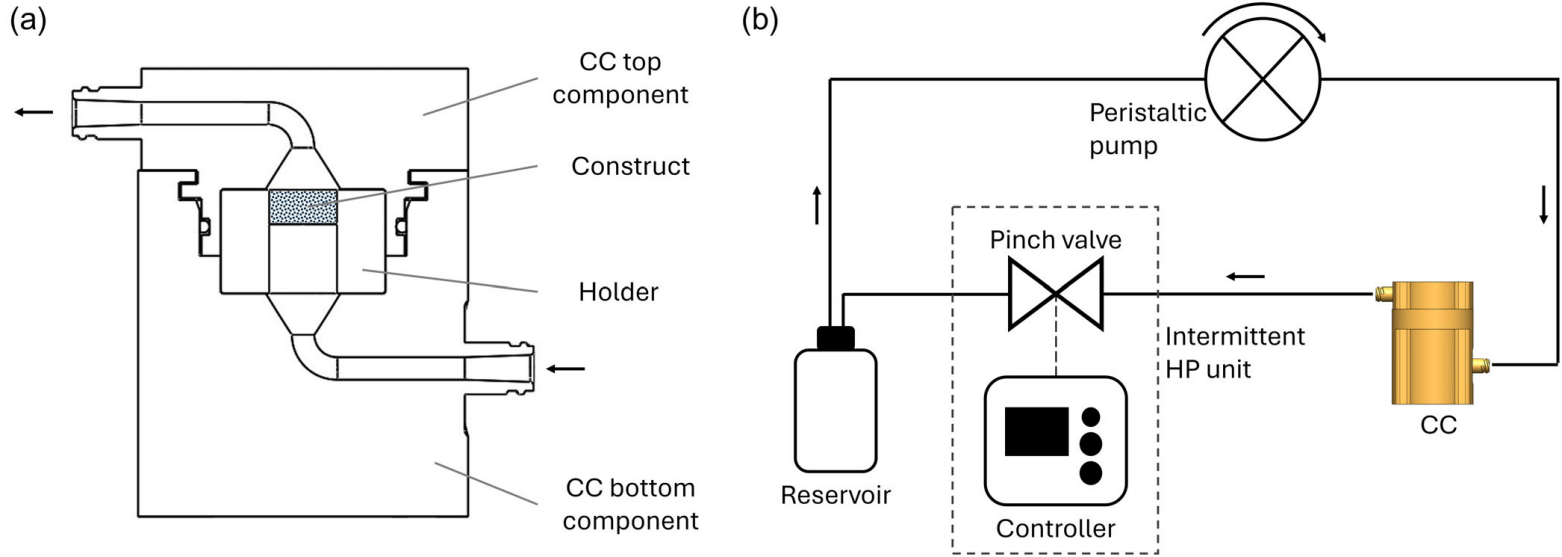
(a) Sectional view of the CC, including its top and bottom components, the holder, the positioning of the construct, the fluidic channels, and the culture medium flow path (black arrows); (b) Schematic representation of the FSS and intermittent HP bioreactor set‐up, including the reservoir, the peristaltic pump, the CC, and the intermittent HP unit, the latter composed of the pinch valve and a dedicated custom controller.

#### 2.1.1 Intermittent HP unit

The intermittent HP unit was developed according to the following design requirements. The unit was required to integrate into the hydraulic circuit and to deliver intermittent HP within the range perceived by cells in bone tissue (3–35 kPa^4^, ^7^, ^8^, ^9^, ^10^), with timing compatible with physiological load‐bearing and muscle activation (0.25–20 s^50^). In addition, the unit was designed to be easy to assemble and disassemble and to impose minimal hindrance for use within standard incubators, while providing a user‐friendly interface for straightforward regulation of operational parameters. Finally, a modular design was adopted to enable versatility and the simultaneous testing of up to three bone tissue models in parallel. Each intermittent HP unit is based on a three‐way solenoid pinch valve (MFLX98302‐46, Masterflex, Cole Parmer, USA), fitted with dedicated silicone tubing (Masterflex, Cole Parmer, USA), positioned downstream of the CC and connected to a custom controller (Figure 1b).

To generate intermittent HP, the pinch valve is operated in normally open mode and periodically actuated to close, transiently blocking downstream flow. In detail, when the peristaltic pump is working and the pinch valve remains open (inactive), the culture medium flows through the CC, exposing the construct to FSS and returning to the reservoir in a continuous recirculating loop. Upon automated activation, the pinch valve closes, blocking the flow downstream of the CC and exposing the construct to progressively increasing HP values until the pinch valve is automatically deactivated.

To secure the pinch valve during operation and maintain it at the same height as the CC outlet, a custom base was designed using SolidWorks (Dassault Systemes, France) and 3D‐printed in polylactic acid (PLA) with a Creality K1 Max printer (Creality, China). For quick connection to the controller, the pinch valve wires terminate with a 3.5 mm jack connector (Figure 2a).

**FIGURE 2:**
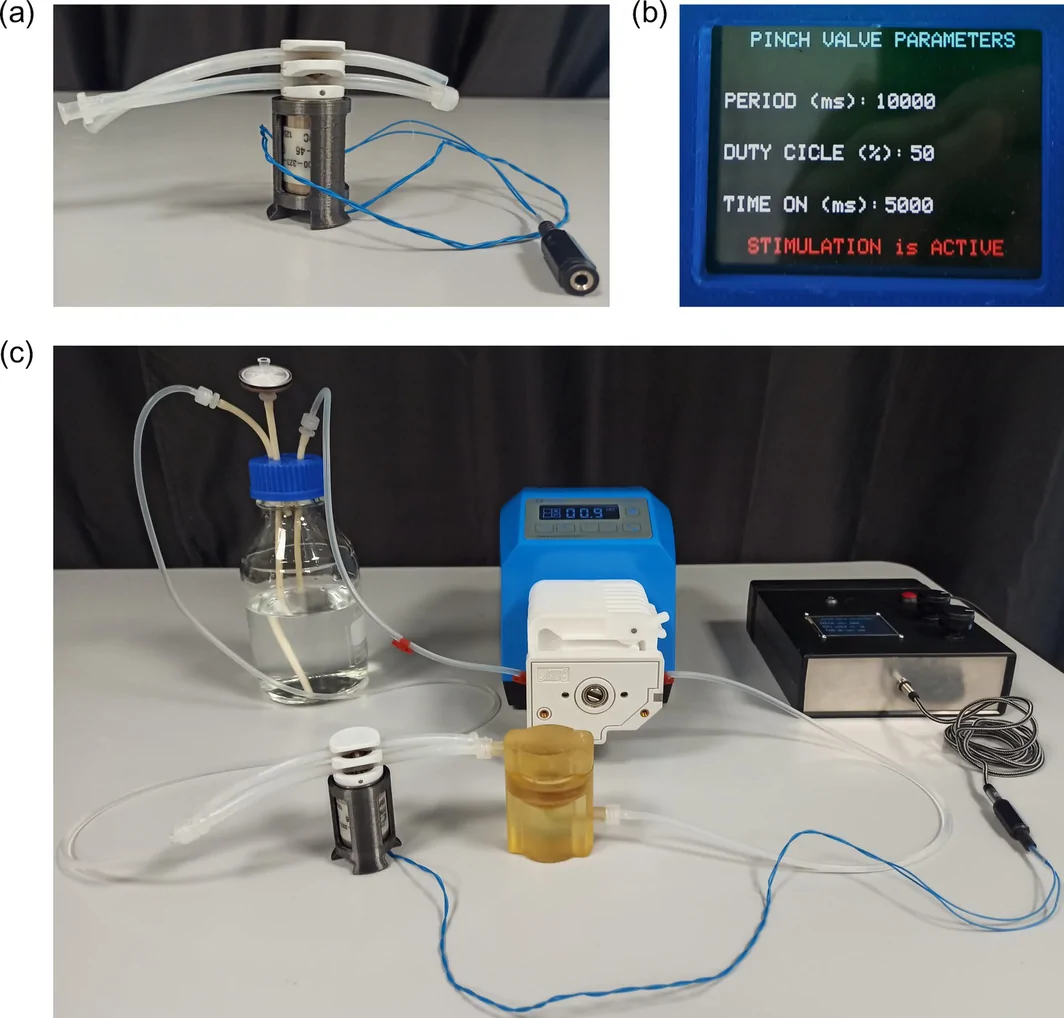
Pictures of the FSS and intermittent HP bioreactor set‐up with a single culture chamber (CC). (a) Pinch valve positioned in the base; (b) LCD screen during stimulation state; (c) Bioreactor set‐up, including the reservoir, the peristaltic pump, the CC, and the intermittent HP unit.

For automated actuation of the pinch valve, a dedicated controller was developed. The controller, embedded in a compact case (145 × 110 × 60 mm^3^), is based on a micro‐controller board (Arduino Micro, Arduino, Italy) that runs purpose‐built software and is equipped with a USB port for software update. The micro‐controller is part of an electric circuit comprising a power supply, an amplification stage, and a user interface. The electric circuits were assembled on RoHS‐compliant printed circuit boards (PCBs), specifically designed by using the open‐source free software KiCAD v7.0.5 and externally manufactured (JLCPCB, China). The power supply is based on two DC/DC converters (TSR1‐2490 and TSR2‐24120, TracoPower, Switzerland), which regulate the voltage from a standard AC/DC adapter connected through a dedicated on/off switch, to supply the micro‐controller (9 V, 1 A) and the amplification stage and pinch valve (12 V, 2 A), respectively. The amplification stage, consisting of two MOSFET transistors (IRLZ44NPBF, Infineon, Germany) and power resistors (10 Ω, 11 W, TE Connectivity, Switzerland), converts the digital signals from the micro‐controller into the driving voltage for the pinch valve while reducing heating. A protective diode prevents high‐voltage spikes that could be generated by the inductive load of the pinch valve. The user interface consists of an LCD display (Adafruit, USA), a status LED, a start/stop button, and two analog potentiometers, allowing activation and deactivation of the pinch valve and setting of the operational parameters. These parameters are the stimulation period (*T*), defined as the total duration of one stimulation cycle and settable between 0.25 and 30 with 0.25 s increments, and the duty cycle (DC), defined as the fraction of each stimulation period during which the valve remains closed (1%–100%, with 1% increments).

Using the potentiometers, the user can select the operational parameters shown on the LCD display menu, which also shows the resulting valve closure time (TIME ON) for user verification purposes. Upon pressing the start/stop button, the controller software activates the stimulation mode, controlling the pinch valve and switching on the status LED. The LCD display shows the selected *T* and DC values, and the TIME ON value (Figure 2b). The high tunability of the operational parameters enables the delivery of HP waveforms with different amplitudes.

Pressing the start/stop button again stops the stimulation and returns the menu to the idle state.

Following the development of the intermittent HP unit, the bioreactor set‐up was assembled with a single CC and used for performance and characterization tests (Figure 2c). Subsequently, for the biological experiments described in Section 2.4, three CCs and three identical hydraulic circuits were assembled, tested, and integrated into the FSS and intermittent HP bioreactor.

### 2.2 Biomimetic scaffolds

To reproduce the complex micro‐architectural features of native bone tissue, biomimetic scaffolds were fabricated by 3D‐printing. Building upon a previously established methodology^51^, ^52^ detailed in the Supplementary Materials, two scaffold types, referred to as PLA600 and P3S3, were designed to replicate trabecular bone architecture characteristics of the human iliac crest^51^ and of the hemimandibula/hemimaxilla,^53^, ^54^, ^55^, ^56^ respectively (Supplementary Figure S1).

The scaffolds were manufactured in PLA (FormFutura BV, The Netherlands) using a Raise 3D N2 printer (Raise 3D Inc., USA). Micro‐tomographic (μ‐CT) analysis was used to characterize the internal structure of the scaffolds and to verify the conformity to the design criteria. The microtomograph Skyscan 1072 (Bruker microCT, Belgium) was used, powering the x‐ray tube at 40 kV and 248 μA, and setting pixel dimensions at 14.65 × 14.65 μm^2^ (corresponding to 20× magnification and 180° rotation with a step of 0.45°). The 3D reconstruction of the samples was performed using the NRecon v1.7.0 software (Bruker microCT, Belgium), and the porosity, trabecular thickness (TT), and trabecular spacing (TS), shown in Table 1, were calculated using CT‐Analyzer v1.16.9.0 (Bruker microCT, Belgium).

**TABLE 1:** Parameters obtained through μ‐CT analysis for PLA600 and P3S3 scaffolds: Porosity, trabecular thickness (TT), and trabecular spacing (TS).

	PLA600	P3S3
Porosity (%)	60.34	44.70
TT (μm)	350	258
TS (μm)	531	284

### 2.3 Bioreactor testing and characterization

#### 2.3.1 Intermittent HP

The performance of the intermittent HP unit was evaluated by assessing the pressure values developing within the CC in the presence of the biomimetic scaffolds. Prior to testing, a two‐step pretreatment was applied to both PLA600 and P3S3 scaffold samples. To reduce their inherent hydrophobicity and improve wettability, the samples were immersed in phosphate‐buffered saline (PBS) solution, which was subsequently aspirated and discarded. Afterwards, each sample was press‐fitted into a PDMS holder and prepared according to the protocol used for cell seeding. Specifically, 170 μL of 5 mg/mL collagen (PureCol EZ Gel solution, Merck, Italy) was mixed with 30 μL of PBS, which served as a substitute for the cell suspension and culture medium. The resulting mixture was gently pipetted onto the sample surface, and the samples were incubated at 37°C for 3 h to allow collagen gelation. Thereafter, the samples were immersed in PBS and maintained in the incubator overnight. Following complete removal of PBS, the sample–holder assembly was transferred into the CC, and the bioreactor was assembled. The hydraulic circuit was filled with deionized water maintained at 37°C, ensuring complete elimination of air bubbles to avoid artifacts during pressure measurements. Pressure values were acquired using a custom measurement system adapted from previous work,^57^ which incorporates an in‐line physiological relative pressure sensor (SP844, HJK Sensoren, Germany) positioned between the CC and the pinch valve to measure the pressure values downstream the CC (Supplementary Figure S2). The sensor output was interfaced with a 24‐bit data acquisition (DAQ) system (NI9237 module connected to a cDAQ9191, National Instruments, USA), which was operated through a custom‐developed software environment with a LabView (National Instruments, USA) graphical user interface enabling real‐time pressure monitoring and data recording.

To explore the range of pressure values achievable varying the operational parameters *T* and DC while minimizing the number of experimental runs, a design of experiments (DOE) approach was employed. Specifically, the boundaries of the parameter space were defined based on biological and technological constraints, limiting the analysis to 1 s< *T* < 30 s and 50%< DC< 70%. A total of 10 independent experimental conditions was tested to characterize the pressure response space, and all experiments were conducted in triplicate. A scaffold sample was prepared for each repetition to ensure experimental consistency. In addition, the sequence of experimental conditions was randomized at each repetition to minimize potential order effects and inter‐experiment correlations. A flow rate (*Q*) of 0.3 mL/min was selected, consistent with the flow rate used for the biological tests (Section 2.4). To account for slow variations in the mean pressure caused by the peristaltic pump operation, pressure values were recorded for 3 min during each test at a sampling frequency (*f*_*s*_) of 2 kHz. The recorded data were subsequently post‐processed using a custom Matlab (Mathworks, USA) script to extract periodic peak pressure values. Results were evaluated in terms of mean, maximum, and minimum peak pressure values (*P*_mean_, *P*_max_, and *P*_min_, respectively).

#### 2.3.2 FSS

To characterize the FSS developing within the biomimetic scaffolds housed in the CC, scaffold permeability was first assessed. Permeability measurements were performed using a custom permeability test bench (PTB), previously described.^57^ For each scaffold type, experiments were conducted on 4 samples, with 3 repetitions per sample, following a previously established protocol.^57^ Details of the testing procedure are reported in the Supplementary Materials. Following permeability characterization, the FSS values (expressed in Pa) developing within the porous PLA600 and P3S3 scaffolds were estimated using the expression derived by Wang and Tarbell.^58^ This formulation, adopted to characterize effective FSS in scaffolds, provides a valid estimation for constructs characterized by permeability values higher than 10^−10^ m^2^ ^59^:

$$\mathrm{FSS}=\frac{4}{\pi }\frac{\mu }{\sqrt{k}}v_{\mathrm{avg}}$$

where FSS is the wall shear stress (Pa), *k* is the scaffold permeability (m^2^), *μ* is the dynamic viscosity of the culture medium (*μ* = 6.89 × 10^−4^ Pa·s at 37°C), and *v*_avg_ is the average velocity of the culture medium within the construct (m/s). To account for the scaffold porosity, *v*_avg_ was evaluated as:

$$v_{\mathrm{avg}}=\frac{Q}{A\cdot \varphi }$$

where *Q* is the flow rate (mL/min), *A* is the scaffold cross‐sectional area (m^2^), and *φ* is the scaffold porosity.

### 2.4 Biological tests

#### 2.4.1 Cell culture conditions

The human telomerase reverse transcriptase (hTERT)‐immortalized BM‐MSCs (clonal line Y201), previously generated by James et al.,^60^ were maintained using basal medium (BM). The latter comprised low‐glucose Dulbecco's modified Eagle's medium (DMEM; 1 g/L, Gibco, Thermo Fisher Scientific, Inc., USA) supplemented with 15% (v/v) fetal bovine serum (FBS, Gibco, Thermo Fisher Scientific, Inc.) and 1% (v/v) penicillin/streptomycin (Gibco, Thermo Fisher Scientific, Inc.). Cell cultures were maintained in a humidified, temperature‐controlled incubator set at 37°C with a 5% CO_2_ atmosphere. For each experiment, cells were passaged upon reaching 80%–90% confluence using enzymatic digestion with trypsin/ethylenediamine tetraacetic acid (trypsin/EDTA, Gibco Thermo Fisher Scientific, Inc.).

#### 2.4.2 Cell seeding and dynamic culture conditions

For the biological tests, PLA600 scaffolds were selected in preference to P3S3 scaffolds as they enabled a broader range of physiologically relevant HP values associated with daily activities (see Figure 4). In detail, BM‐MSCs were seeded onto PLA600 scaffolds housed within the PDMS holders using a cell‐releasing 0.5% collagen hydrogel by resuspending 2.5 × 10^6^ cells in 30 μL of BM and 170 μL of 5 mg/mL collagen (PureCol EZ Gel solution, Merck, Italy). Following a 4 h incubation period to facilitate cell infiltration and adhesion, the cell‐seeded scaffolds were submerged in BM and incubated overnight. Subsequently, the constructs were cultured for 7 days under the following conditions: (S) static culture in a 12‐well plate; (DP) continuous direct perfusion (*Q* = 0.3 mL/min) within the bioreactor; and (DP + IP) continuous direct perfusion (*Q* = 0.3 mL/min) combined with intermittent HP (*T* = 10 s and *DC* = 50%, resulting in *P*_mean_ = 5.27 kPa, as measured in bioreactor characterization tests; see Table 3) within the bioreactor. Samples were evaluated in triplicates (*n* = 3, Figure 3).

**FIGURE 3:**
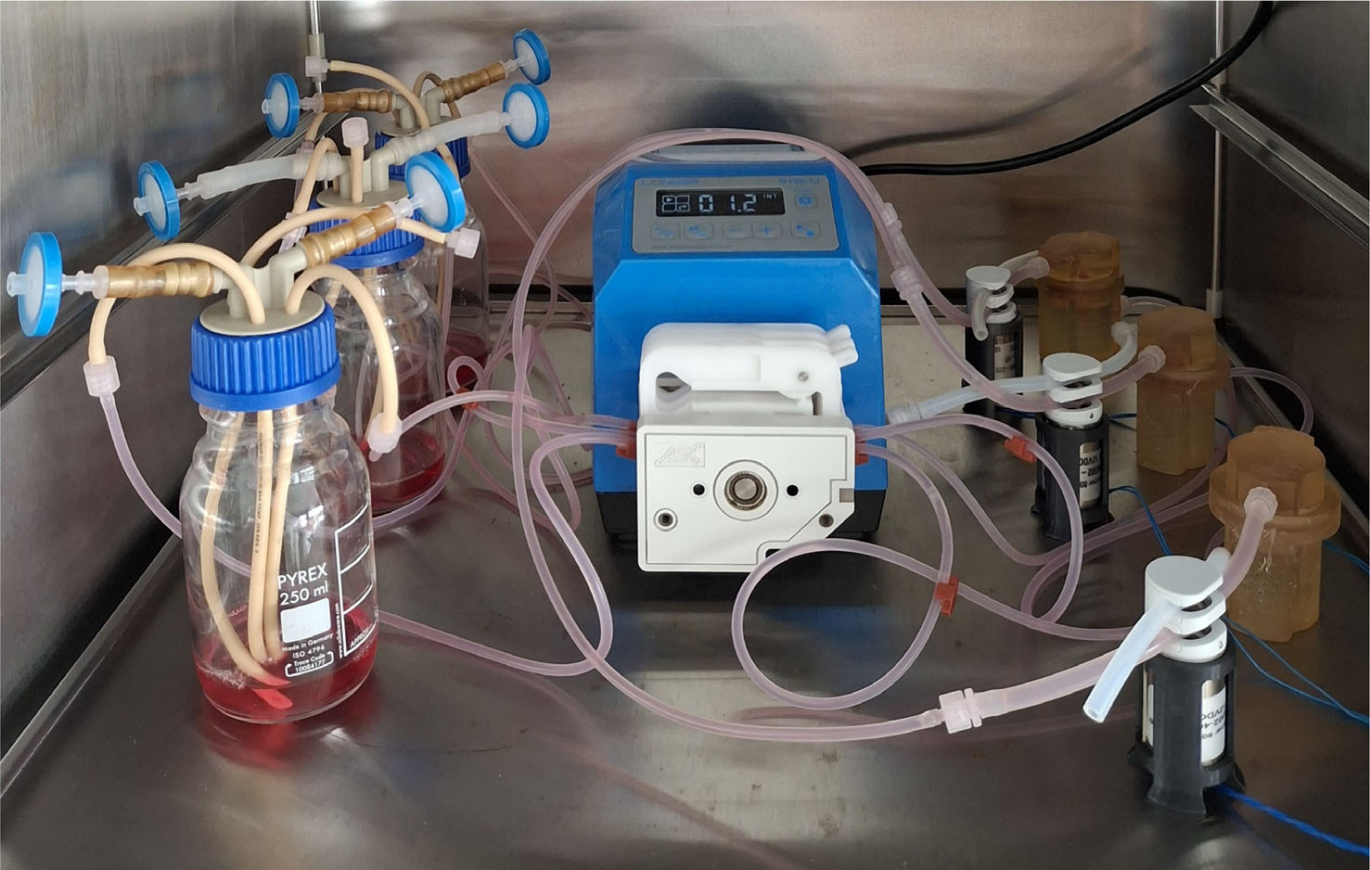
Set‐up of the parallelized, automated FSS and intermittent HP bioreactor within the incubator.

#### 2.4.3 Cell viability assessments: Resazurin reduction assay and live/dead assay

The metabolic activity of BM‐MSCs was assessed using the resazurin reduction assay. A 1% resazurin stock solution was prepared by dissolving resazurin sodium salt (Merck, Darmstadt, Germany) in 1× PBS and filtering it. This stock solution was then diluted in the BM to a final concentration of 0.015% (v/v) immediately before the assay. Briefly, on day 7 of culture, constructs were carefully removed from the bioreactor CCs and transferred to a 12‐well cell culture plate. To each well, 3 mL of the resazurin working solution was added. Following 3 h of incubation, 100 μL aliquots of the supernatant were collected, and fluorescence was quantified using a microplate reader (Tecan GENios Microplate Reader, Spark, Tecan Trading AG, Switzerland) at excitation and emission wavelengths of 530 nm and 590 nm, respectively. Cell viability was also evaluated on day 7 of culture using the LIVE/DEAD™ Viability/Cytotoxicity Kit (Invitrogen Life Technologies, Italy). Briefly, on day 7 of culture, constructs were carefully removed from the bioreactor CCs and transferred to a 24‐well cell culture plate. The constructs were then incubated for 15 min at 37°C with 1 mL of BM containing 2 μM calcein‐AM and 2 μM ethidium homodimer‐1 (EthD‐1). Subsequently, cells were visualized using a fluorescence microscope (THUNDER Imager 3D Cell Culture, Leica Microsystems, Italy).

#### 2.4.4 Cell infiltration and distribution assessments

Cell infiltration and morphology were assessed on day 7 using scanning electron microscopy (SEM; JSM‐IT500, JEOL, Italy). For SEM analysis, constructs were removed from the bioreactor CCs, placed in a 24‐well plate, and gently washed with PBS (1×). Fixation was performed overnight at 4°C using 2.5% glutaraldehyde. Subsequently, the constructs were washed twice with PBS (1×) before dehydration. Dehydration involved a graded ethanol series (70%, 90%, and 100%, twice) with 20‐min incubations at each concentration. Samples were then treated with hexamethyldisilazane (HMDS) for 30 min, air‐dried, and sputter‐coated with gold (e.g., using a JEOL Smart Coater, Italy) before imaging.

#### 2.4.5 Gene expression analysis

The expression of osteogenic genes was assessed using reverse transcription‐quantitative PCR (RT‐qPCR). Total RNA was extracted from constructs using TRIzol™ Reagent (Invitrogen Life Technologies, Italy) following the manufacturer's protocol, with an extended cell lysis step of 10 min. RNA yield and quality were determined spectrophotometrically using a NanoDrop One (Thermo Fisher Scientific Inc., Italy). Subsequently, 1 μg of total RNA was reverse transcribed using the Verso cDNA Synthesis Kit (Thermo Fisher Scientific, Inc., Italy) on a Mastercycler X50s (Eppendorf AG). qPCR was performed using 1 ng/μL of cDNA, SYBR™ Green Universal Master Mix (Applied Biosystems, Thermo Fisher Scientific, Inc., Italy), and gene‐specific primers at a final concentration of 2.5 μM. Reactions were run and monitored on a CFX96™ Real‐Time System (Bio‐Rad Laboratories, Italy) according to the manufacturer's standard cycling conditions. Cycle threshold (*Cq*) values for target genes were normalized to the reference gene, Tyrosine 3‐Monooxygenase/Tryptophan 5‐Monooxygenase Activation Protein Zeta (YWHAZ), and relative gene expression was quantified using the 2^−ΔΔCq^ method^61^, ^62^ relative to the control group (continuous direct perfusion [DP]). Primer sequences for all analyzed genes are detailed in Table 2. The analysis was performed for both DP and DP + IP experimental conditions.

**TABLE 2:** Description of the designed primers.

Gene	Forward primer	Reverse primer
Tyrosine 3‐Monooxygenase/Tryptophan 5‐Monooxygenase Activation Protein Zeta (YWHAZ)	5′‐GGA GGG TCG TCT CAA GTA T‐3′	5′‐ATC TCT TAG CTC CGT CTC AA‐3′
Runt‐related transcription factor 2 (RUNX2)	5′‐GAA TGC CTC TGC TGT TAT GA‐3′	5′‐GAA GAC GGT TAT GGT CAA GG‐3′
Alkaline phosphatase (ALP)	5′‐GAG TAT GAG AGT GAC GAG AAA G‐3′	5′‐GAA GTG GGA GTG CTT GTA TC‐3′
Osteopontin (OPN)	5′‐CCC ATC TCA GAA GCA GAA‐3′	5′‐TGG CTT TCG TTG GAC TTA C‐3′
Osteocalcin (OCN)	5′‐CAG GCG CTA CCT GTA TCA A‐3′	5′‐CCG ATA GGC CTC CTG AAA G‐3′
Collagen Type I Alpha 1 Chain (COL1A1)	5′‐GGA TTC CAG TTC GAG TAT GG‐3′	5′‐CAG TGG TAG GTG ATG TTC TG‐3′
SRY‐box transcription factor 9 (SOX9)	5′‐TCT GAA CGA GAG CGA GAA‐3′	5′‐GCG GCT GGT ACT TGT AAT C‐3′
Marker of Proliferation Ki‐67 (Ki‐67)	5′‐GGA AAG TGG ACG TAG AAG AAG‐3′	5′‐GCA CTG GAG TTC CСА ТАА АТ‐3′
Collagen Type II Alpha 1 Chain (COL2A1)	5′‐ACG ATG GGC AGA GGT ATA AT‐3′	5′‐CAG ATT ATG TCG TCG CAG AG‐3′

#### 2.4.6 Protein analysis

Total proteins were extracted using the TRIzol™ Reagent (Invitrogen Life Technologies, Italy), following the manufacturer's instructions. Then, protein concentrations were quantified using the Bradford assay with the Bradford dye reagent, ready‐to‐use solution (Thermo Fisher Scientific, Inc.). Briefly, the standards were prepared by diluting bovine serum albumin (BSA, Thermo Fisher Scientific, Inc.) to a concentration range of 200–1600 μg/mL. Each standard and test sample (30 μL) was added per cuvette, followed by 1.5 mL of Bradford reagent. The cuvettes were incubated at room temperature, protected from light for 10 min, and then absorbance was measured at 595 nm using a UV–vis Spectrophotometer (Implen NanoPhotometer®, Germany). Protein concentrations were calculated using the calibration curve.

For protein profile evaluation, equal volumes (25 μL) of the prepared protein samples were subjected to sodium dodecyl sulfate‐polyacrylamide gel electrophoresis (SDS‐PAGE). Electrophoretic separation was performed on a 12.5% Tris‐glycine SDS‐PAGE gel at a constant voltage of 150 V for 1 h. Following separation, the gel was imaged to evaluate the protein profile of the samples via Coomassie Brilliant Blue (Thermo Fisher Scientific, Inc.).

#### 2.4.7 Statistical analysis

All biological experiments were run with three biological replicates. Continuous variables were compared by the two‐tailed *t*‐test, and statistical significance was defined as a *p*‐value <0.05. The analysis was conducted using R,^63^ RStudio,^64^ and the tidyverse package.^65^

## 3 RESULTS

### 3.1 Bioreactor testing and characterization

#### 3.1.1 Intermittent HP

The mean, maximum, and minimum peak pressure values (*P*_mean_, *P*_max_, *P*_min_, respectively) developing within the collagen‐loaded PLA600 and P3S3 scaffolds are reported in Table 3. Owing to the wide range of testing conditions, the *P*_mean_ values spanned approximately one order of magnitude (Figure 4) and were higher for P3S3 scaffolds (*P*_mean_ = 4.53–30.59 kPa) compared with PLA600 scaffolds (*P*_mean_ = 1.81–15.09 kPa), consistent with the lower permeability of the P3S3 architecture, as reported in Section 3.1.2. Overall, for both scaffold types, the *P*_mean_ values and the corresponding *P*_min_ − *P*_max_ ranges measured under different operational conditions effectively replicated native pressure ranges associated with daily activity (~3–8 kPa) and intense activity (~10–35 kPa). Considering the full range of peak pressure values measured, *T* = 10 s and DC = 50% were identified as the optimal operational parameters for biological experiments performed using BM‐MSC‐seeded PLA600 scaffolds. Indeed, these parameters yielded a *P*_mean_ of 5.27 kPa and a peak pressure range (*P*_min_ = 2.25 kPa, *P*_max_ = 7.84 kPa) closely matching pressure levels associated with in vivo daily activity condition.

**TABLE 3:** Mean, minimum, and maximum peak pressure values for PLA600 and P3S3 scaffolds, respectively.

*T*, DC	PLA600			P3S3		
	*P*_mean_ (kPa)	*P*_min_ (kPa)	P_max_ (kPa)	*P*_mean_ (kPa)	*P*_min_ (kPa)	*P*_max_ (kPa)
1 s, 50%	1.81	0.99	2.15	4.53	3.38	5.23
1 s, 70%	2.05	1.10	2.49	4.96	3.43	6.02
5 s, 50%	3.38	1.21	4.70	7.69	3.43	10.67
5 s, 70%	4.12	1.69	6.04	9.34	3.60	13.55
10 s, 50%	5.27	2.25	7.84	11.74	5.66	17.12
10 s, 70%	6.58	3.88	9.80	14.90	8.20	21.07
15 s, 70%	7.58	6.62	9.05	19.95	13.30	22.19
20 s, 70%	10.24	8.35	11.60	24.63	20.39	29.82
25 s, 70%	12.83	9.94	15.52	27.68	19.81	35.42
30 s, 70%	15.09	12.83	17.60	30.59	22.41	36.92

**FIGURE 4:**
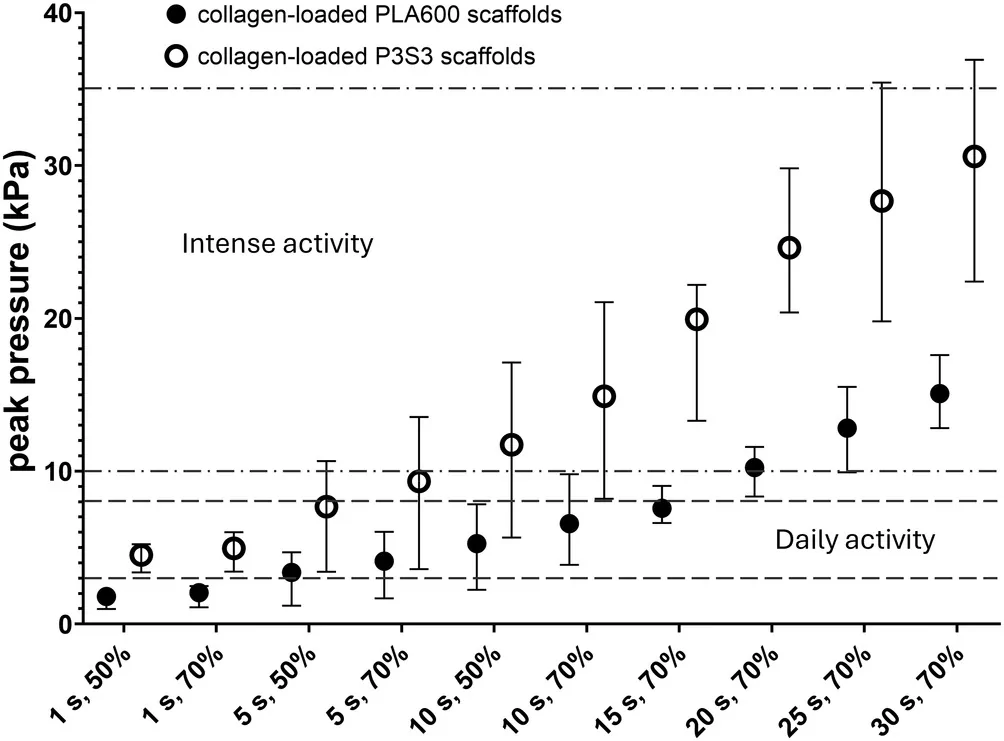
Peak pressure values measured in collagen‐loaded PLA600 and P3S3 scaffolds under different operational conditions, reported as *P*_mean_ values and corresponding *P*_min_ − *P*_*max*_ ranges.

#### 3.1.2 FSS

The mean permeability values measured for PLA600 and P3S3 scaffolds were 2.36 × 10^−10^ m^2^ and 2.14 × 10^−10^ m^2^, respectively.

Consistently, the FSS values generated within the two scaffold types over the range of flow rates provided by the pump (*Q* = 0.024–24 mL/min) were calculated analytically, yielding the values reported in Table 4. In particular, at *Q* = 0.3 mL/min, the FSS values developing within the scaffolds were 6.02 mPa for PLA600 scaffolds and 8.54 mPa for P3S3 scaffolds.

**TABLE 4:** Range of FSS values developing within PLA600 and P3S3 scaffolds, respectively.

Flow rate (mL/min)	FSS (mPa)	
	PLA600	P3S3
0.024	0.48	0.68
24	481.99	683.26

### 3.2 Biological tests

#### 3.2.1 Cell viability, infiltration, and distribution assessments

Cell viability of BM‐MSCs seeded within PLA600 scaffolds and cultured under different conditions was assessed after 7 days (Figure 5). As demonstrated by the resazurin reduction assay (Figure 5a), culturing the constructs within the bioreactor, either under DP alone or combined with intermittent HP (DP + IP) simulating daily activity, significantly promoted cell metabolic activity compared with static culture (S) (*p*< 0.05). Notably, no significant differences in metabolic activity were observed between the DP and DP + IP conditions, indicating that cell viability was preserved within the bioreactor under both DP alone and DP combined with intermittent HP. The live/dead assay (Figure 5b) further supported these findings, as green‐stained cells (indicating live cells) were predominant across all experimental conditions, suggesting a high density of viable cells. Furthermore, the images acquired at different *z*‐coordinates (representing both the scaffold surface and the inner core) within the constructs confirmed that bioreactor‐based culture effectively supported a homogenous cell distribution throughout the constructs. Since the background green fluorescence emitted by live cells through the entire PLA600 scaffolds can partially obscure fine cellular details during fluorescence imaging, high‐magnification analysis was performed to better distinguish the various cellular structures from the scaffold's matrix. As shown in Supplementary Figure S3, there is a coexistence of dense cell sheets stretching across the pores of the scaffold alongside individual adherent cells. Furthermore, SEM imaging was utilized as a complementary approach, which successfully corroborated our fluorescence findings. SEM micrographs confirmed the optimal cell density, infiltration, and uniform distribution throughout the scaffolds under different experimental conditions (Figure 6).

**FIGURE 5:**
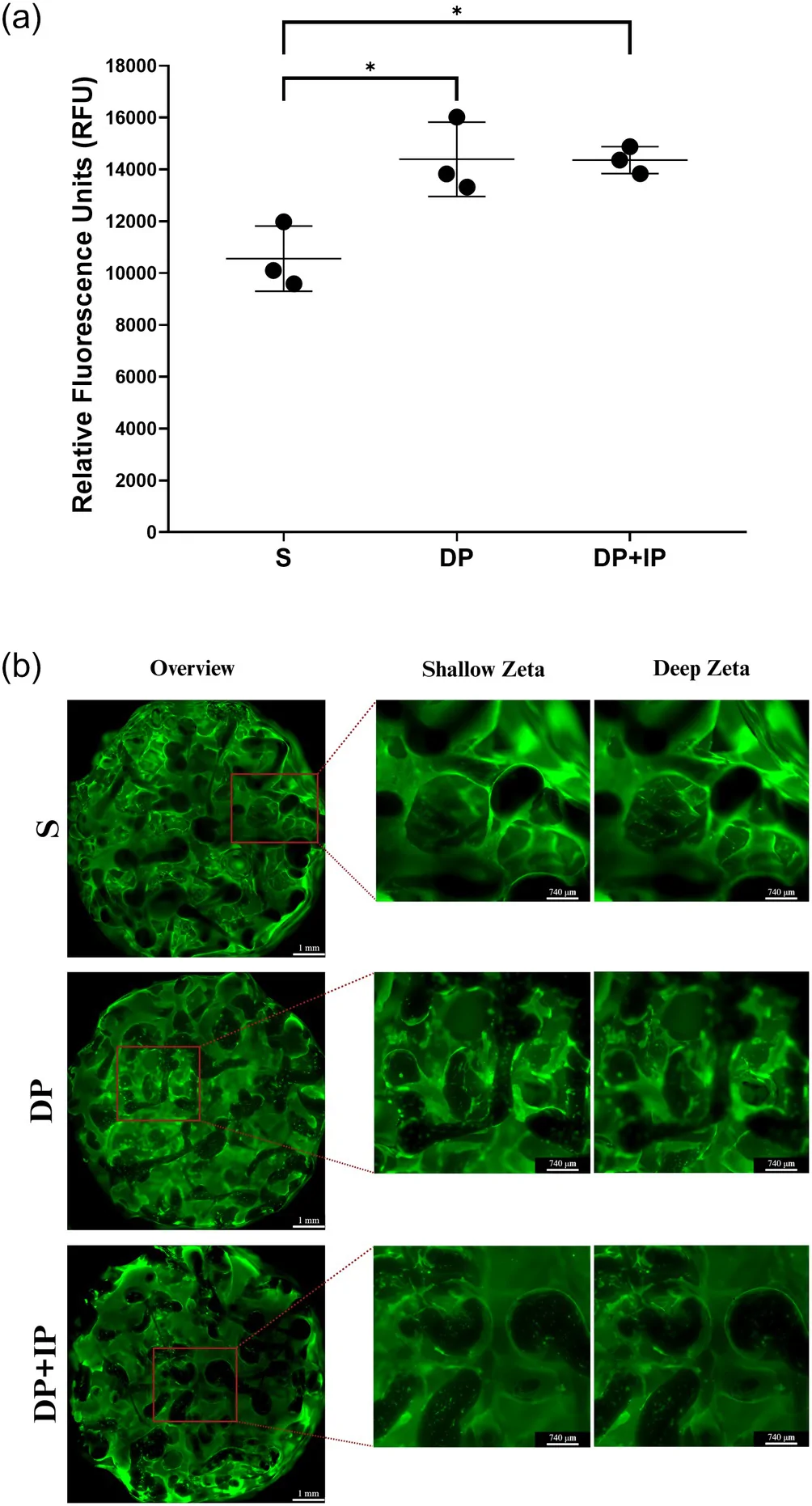
Cell viability of BM‐MSCs seeded within PLA600 scaffolds and cultured for 7 days under static culture (S), direct perfusion (DP), or direct perfusion combined with intermittent HP (DP + IP) simulating daily activity. (a) Resazurin reduction assay results. Dots represent the quantification of three biological replicates, and error bars indicate the mean and the standard deviation (*, *p*< 0.05). (b) Live/dead assay showing a cross‐section overview of the scaffolds (scale bar: 1 mm) and distinct views of a specific region of interest at different *z*‐coordinates within the 3D constructs (shallow: Near the scaffold surface, scale bar: 740 μm; deep: Toward the scaffold center, scale bar: 740 μm).

**FIGURE 6:**
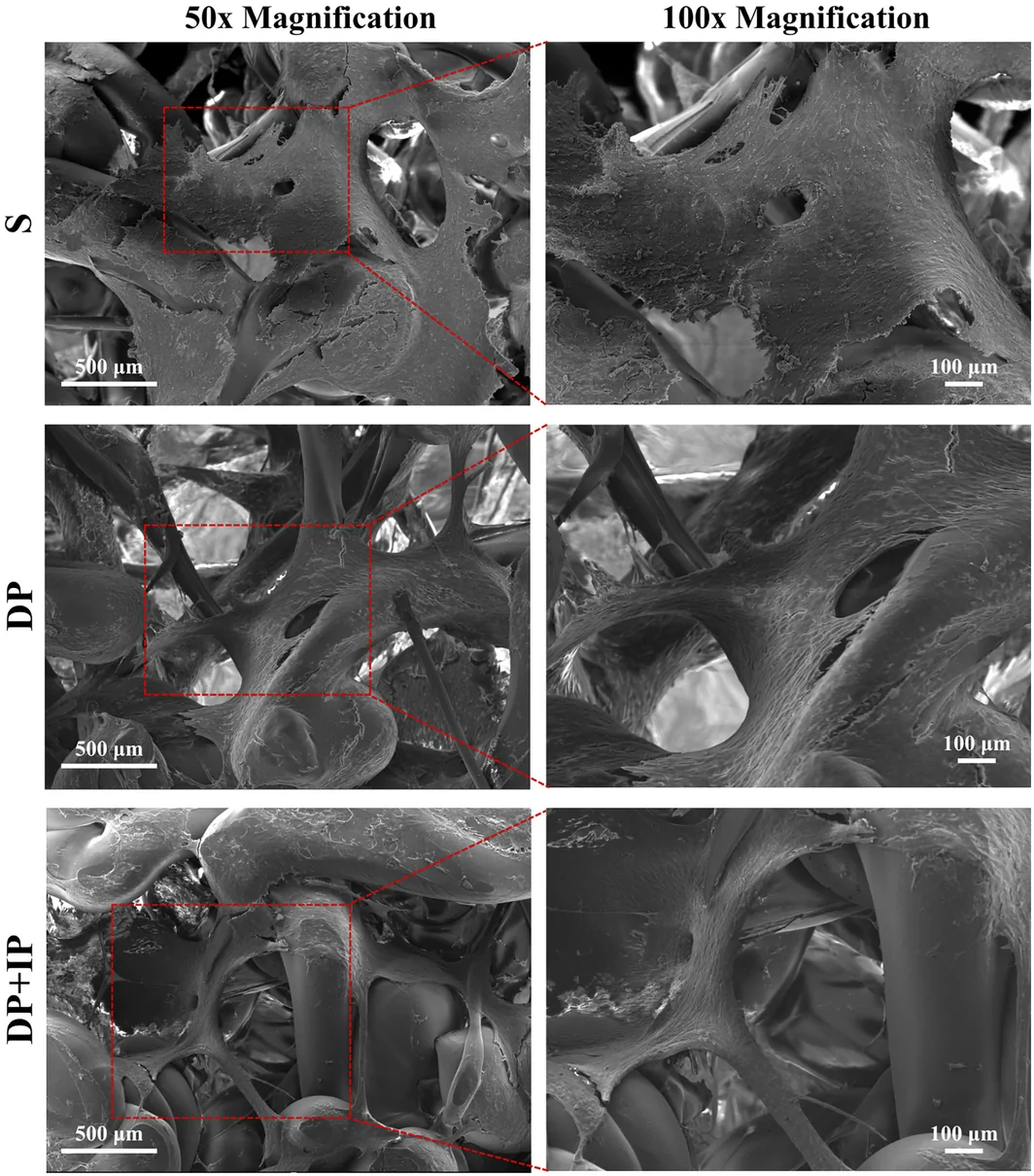
SEM images showing the morphology of BM‐MSCs seeded within PLA600 scaffolds and cultured under static culture (S), direct perfusion (DP), or direct perfusion combined with intermittent HP (DP + IP) simulating daily activity. The left column presents low‐magnification overview images of the cell‐seeded scaffolds (50×, scale bar 500 μm), while the right column shows higher‐magnification views of representative regions of interest (100×, scale bar 100 μm).

#### 3.2.2 Gene expression analysis

Gene expression levels of key osteogenic markers (RUNX2, ALP, OPN, OCN, and COL1A1) were analyzed using the 2^−ΔΔ^ *Cq* method to compare the DP + IP group with the DP group, which was treated as the control (Figure 7). The analysis revealed a notable upregulation of ALP expression in the DP + IP group, with an approximately 2.7‐fold increase compared with the DP group. Furthermore, COL1A was upregulated by 1.4‐fold, while OPN exhibited a modest ~1‐fold increase under DP + IP conditions. In contrast, RUNX2 and OCN showed limited differences between the two experimental conditions. Despite the absence of a uniform upregulation across all investigated osteogenic genes, the observed 2.7‐fold increase in ALP expression and 1.4‐fold increase in COL1A1 are considered biologically relevant, particularly given the relatively short 7‐day culture period. Indeed, as ALP is an early marker of osteoblast differentiation, these upregulations suggest a positive influence of combined direct perfusion and intermittent HP stimulation simulating daily activity on early osteogenic processes.

**FIGURE 7:**
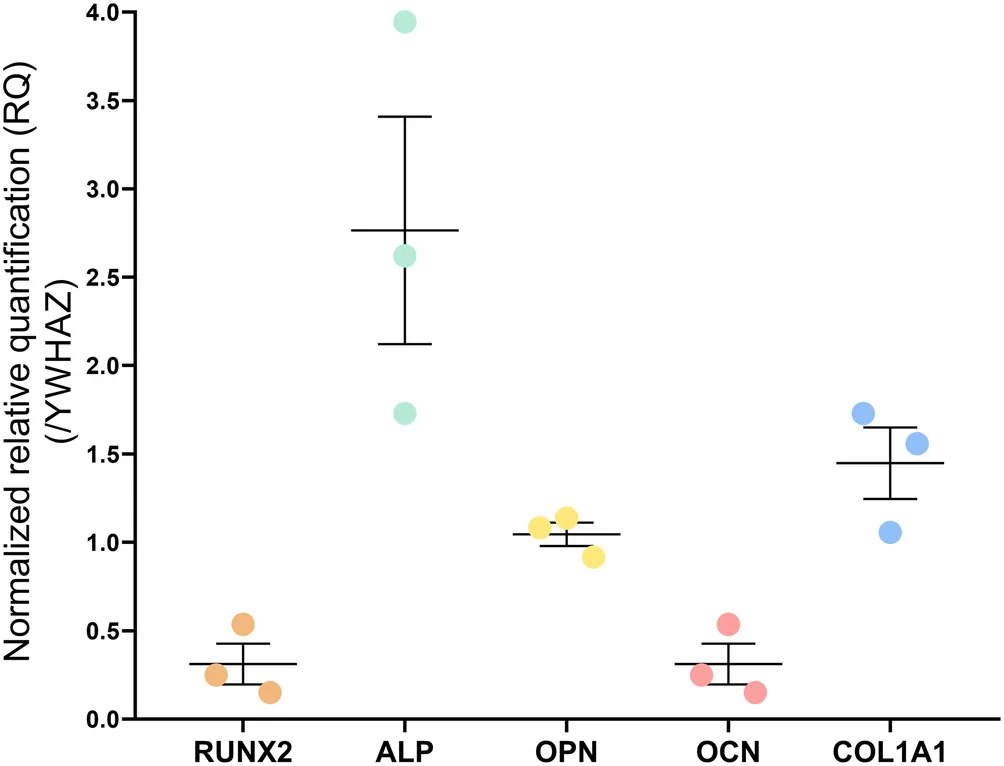
Gene expression analysis of key osteogenic markers (RUNX2, ALP, OPN, OCN and COL1A1) in BM‐MSCs seeded within PLA600 scaffolds and cultured for 7 days under different conditions within the bioreactor. The DP + IP group (direct perfusion combined with intermittent HP simulating daily activity) was compared to the DP group (direct perfusion alone) using the 2^−ΔΔ*Cq*^ method. Dots represent the relative quantification of three biological replicates, and error bars indicate the mean and the standard error of the mean (s.e.m.).

To further characterize the phenotypic lineage and proliferative behavior of the cells under the combined mechanical stimulation, our analysis was expanded to evaluate chondrogenic differentiation (SOX9 and COL2A1) and cellular proliferation (Ki‐67) (Supplementary Figure S4). Our results revealed that SOX9 expression exhibited less than a 1‐fold change, while COL2A1 expression was increased by 1 fold in the DP + IP group compared to the DP group. This lack of upregulation demonstrates that the combined continuous perfusion and intermittent HP (DP + IP) does not induce chondrogenesis, which can be further confirmed by the COL1A1/COL2A1 ratio. In contrast, expression of the proliferation marker Ki‐67 was significantly upregulated by approximately 1.6‐fold in the DP + IP group relative to the DP group. This may suggest that the applied HP profile (*T* = 10 s, DC = 50%, *P*_mean_ = 5.27 kPa) acts as a biophysical stimulus that can enhance cellular proliferation during the early stages of culture.

#### 3.2.3 Protein analysis

To ensure that equal amounts of high‐quality protein were utilized for the electrophoretic separation, the Bradford assay was conducted. Samples demonstrated consistent and robust total protein concentrations across both groups, as shown in Table 5.

**TABLE 5:** Total protein concentration of DP and DP + IP group via Bradford assay.

Sample ID	Concentration (μg/mL)
DP_1	1160
DP_2	1390
DP_3	1350
DP + IP_1	1390
DP + IP_2	940
DP + IP_3	1250

To evaluate early‐stage macromolecular changes, total protein profiles were obtained using SDS‐PAGE followed by Coomassie Blue staining. As shown in Figure 8, both the control (DP) and experimental (DP + IP) groups exhibited well‐defined, distinct bands spanning a broad molecular weight range between ~10 and ~250 kDa, which confirms protein stability and a lack of degradation during extraction. Furthermore, the consistent band intensities observed across all lanes validate the accuracy of the Bradford quantification and ensure that equal concentrations of total protein were uniformly loaded.

**FIGURE 8:**
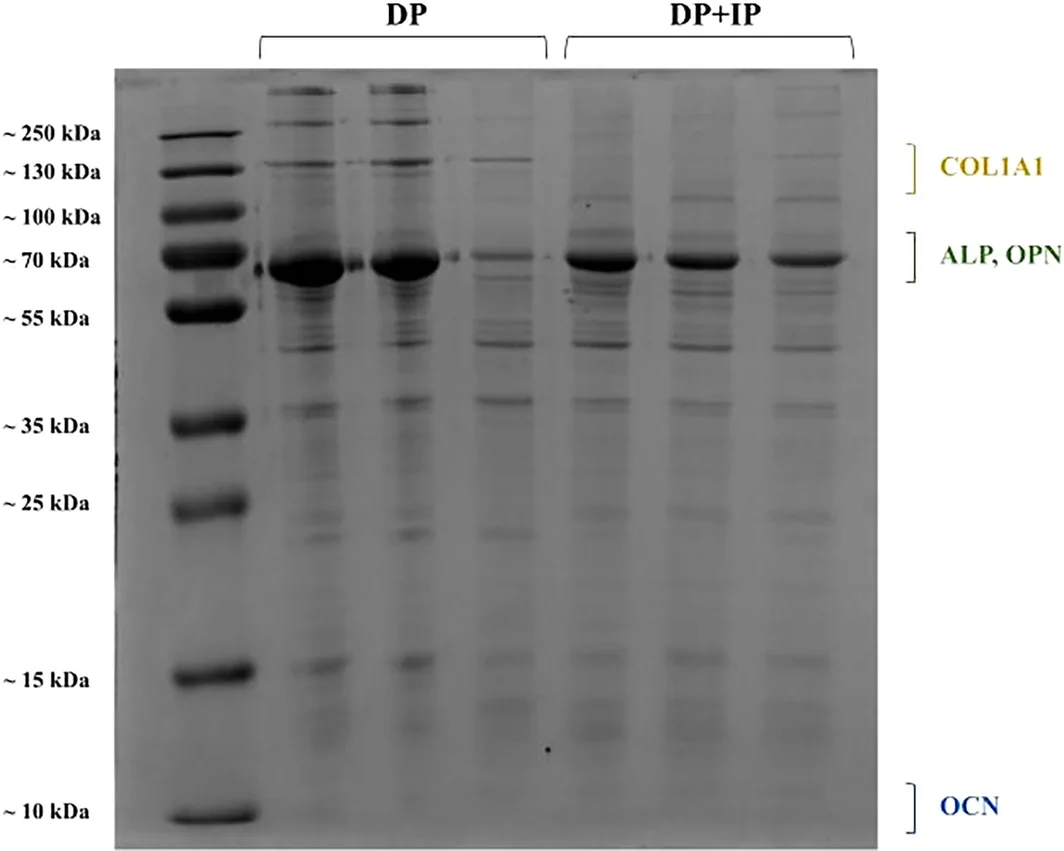
Early‐stage macromolecular protein profile at day 7 of osteogenic differentiation. SDS‐PAGE (Coomassie Blue stained) of total cellular lysates from DP and DP + IP groups at day 7. The profile demonstrates sample integrity and consistent total protein loading across groups, aligning with early‐stage proteomic shifts before peak translation of late‐stage osteogenic matrix markers. Bands are labeled according to the expected molecular weights of key osteogenic markers (COL1A1, ALP, OPN, and OCN).

Interestingly, both groups displayed a highly intense band at approximately 70 kDa. This major band represents a combination of serum albumins and structural proteins,^66^ as well as other early osteogenic markers, including ALP protein, which typically migrates as a monomer in the 70–80 kDa range.^67^ Overall, the protein profiling at day 7 reflected broader, early‐stage proteomic shifts characterized by overlapping bands, which aligns with the expected biological timeline of osteogenic differentiation. While gene expression analyses demonstrated approximately a 2.7‐fold increase in transcriptional upregulation of ALP, the translation and extracellular deposition of mature, heavy bone matrix proteins typically do not peak until later time points.^68^ Thus, future studies will incorporate targeted or pan protein‐level analyses to validate whether transcriptional upregulations translate into increased osteogenic protein expression.

## 4 DISCUSSION

The regeneration and maintenance of bone tissue are tightly regulated by its complex mechanical microenvironment,^16^ with variations in FSS and HP representing two principal cues influencing stem cell fate and bone remodeling.^69^ Although the individual effects of FSS and HP stimuli have been widely studied, their combined impact and their interaction within a physiologically relevant 3D bone tissue model remain poorly investigated and understood.^20^

To gain deeper insight into the synergistic mechanotransduction mechanisms involved in osteogenesis, in this study we developed and tested a parallelized, automated bioreactor capable of applying combinations of FSS and intermittent HP stimulations to 3D bone tissue models. The platform builds on a previously developed direct perfusion bioreactor for controlled FSS stimulation of 3D constructs,^24^ which was upgraded through the integration of a modular and automated intermittent HP unit. This unit, based on a solenoid pinch valve actuated by a custom controller, enables the generation of tunable and physiologically relevant pressure waveforms, while maintaining compatibility with standard incubators and ensuring ease of assembly and use. Most existing bioreactors applying intermittent HP to 3D bone tissue models operate without medium recirculation or perfusion,^37^, ^38^, ^40^, ^41^ thereby limiting their ability to reproduce the dynamic mechanical and transport conditions of native bone. Conversely, modular bioreactors designed to deliver both FSS and cyclic HP typically restrict stimulation to a single mechanical cue at a time^45^, ^46^ or are limited to 2D monolayer cultures.^47^, ^48^ Thus, the key novelty of the proposed bioreactor lies in the integration of direct perfusion with intermittent HP within the same device, enabling the combined application of these stimuli to 3D bone tissue models to more closely approximate the complexity of the native bone mechanical microenvironment. Furthermore, the wide tunability of the operational parameters of both the peristaltic pump (*Q* = 0.024–24 mL/min) and the intermittent HP unit (*T* = 0.25–30 s, DC = 1%–100%) allows the implementation of multiple stimulation protocols, which can be tailored to specific bone tissue models and experimental objectives. Finally, the presence of three independent CCs with dedicated hydraulic circuits enables parallel culture under specific combinations of FSS and HP stimulations, thereby increasing experimental throughput and reproducibility.

Once the FSS and intermittent HP bioreactor was developed, characterization tests were performed using two different biomimetic PLA scaffold types (PLA600 and P3S3) designed to replicate the trabecular architecture of human bone. Specifically, to assess the performance of the intermittent HP unit, the scaffold samples were pretreated with PBS and infiltrated with collagen gel following the same protocol used for cell seeding, incubated, and subsequently transferred to the bioreactor CC for pressure measurements. The characterization over a wide range of values of the pinch valve operational parameters (*T* and DC) confirmed the reliability and versatility of the intermittent HP unit. Indeed, mean peak pressure values measured on collagen‐loaded PLA600 scaffolds (*P*_mean_ = 1.81–15.09 kPa, Table 3) and collagen‐loaded P3S3 scaffolds (*P*_mean_ = 4.53–30.59 kPa, Table 3) were consistent with physiological intraosseous pressure levels observed during daily activities (~3–8 kPa) and intense loading conditions (~10–35 kPa).

To estimate the FSS generating within the scaffolds, we first measured their permeability. The obtained permeability values (2.36 × 10^−10^ m^2^ for PLA600 scaffolds and 2.14 × 10^−10^ m^2^ for P3S3 scaffolds) confirmed the biomimetic nature of the scaffold architectures, as they are consistent with permeability values reported in the literature for human trabecular bone (10^−8^–10^−11^ m^2^).^70^, ^71^, ^72^, ^73^ Regarding FSS characterization, the analytical approach demonstrated that the FSS ranges achievable using PLA600 scaffolds (0.48–481.99 mPa, Table 4) and P3S3 scaffolds (0.68–683.26 mPa, Table 4) encompass the FSS range (0.55–24 mPa) reported to promote in vitro osteogenic differentiation of hMSCs and increased extracellular matrix mineralization in 3D bone tissue models.^74^, ^75^, ^76^, ^77^

Overall, the characterization of the HP and FSS stimuli across different scaffold types confirmed the versatility of the bioreactor for culturing diverse 3D bone tissue models, as well as its ability to deliver a wide range of physiologically relevant mechanical stimuli capable of supporting different stages of bone tissue culture and maturation.

Finally, we investigated the impact of combined FSS and HP stimulation on early osteogenic processes in specific 3D bone tissue models. BM‐MSCs were seeded within collagen‐loaded PLA600 scaffolds, selected for their ability to cover pressure values typical of daily activities, and cultured in the bioreactor for 7 days. The experimental conditions included static culture (S), continuous direct perfusion (DP), and continuous direct perfusion combined with intermittent HP (DP + IP). The flow rate was set equal to 0.3 mL/min, corresponding to an estimated FSS of 6.02 mPa. This value falls within the range reported to support osteogenic differentiation in vitro and is approximately one order of magnitude lower than FSS values shown to induce cell detachment or death (>60 mPa).^78^ The characterization tests supported the selection of the pinch valve parameters for the DP + IP condition. Specifically, *T* = 10 s and DC = 50% were identified as suitable for delivering to the 3D bone tissue models a mean peak pressure (*P*_mean_ = 5.27 kPa) and a peak pressure range (*P*_min_ = 2.25 kPa, *P*_max_ = 7.84 kPa) comparable to those experienced during in vivo daily activities. Given the short culture duration and the resulting early stage of tissue development, these moderate pressure values were intentionally selected to avoid overstimulation of the constructs, as the biological investigation was focused on early osteogenic responses rather than on later‐stage maturation, which would require higher mechanical loads.

The biological results demonstrated that dynamic culture conditions (DP and DP + IP) enhanced the viability, infiltration, and distribution of BM‐MSCs within the PLA600 scaffolds compared to static culture (S). These findings, supported by resazurin reduction, live/dead staining, and SEM analysis, align with previous studies showing that mechanical stimulation is critical for promoting cell survival and integration in 3D bone tissue models.^79^, ^80^ At the molecular level, the combined application of FSS and HP stimulation in the DP + IP group triggered a 2.7‐fold upregulation of the early osteogenic marker ALP compared to the perfusion‐only DP condition, suggesting that the combination of continuous perfusion with intermittent HP accelerates early osteogenic processes. These results agree with the findings of Zhou et al.,^48^ who developed a bioreactor for concurrently exposing BM‐MSCs to FSS and cyclic HP, and reported significantly increased expression of ALP after 7 days of combined stimulation. In addition, building on the study from Stavenschi et al.,^46^ which demonstrated that physiological levels of pressures, as low as 10 kPa, can drive hBMSC osteogenic lineage commitment, we observed the acceleration of early osteogenic processes on 3D constructs based on hBMSCs when applying lower intermittent pressures (*P*_min_ = 2.25 kPa, *P*_max_ = 7.84 kPa, *P*_mean_ = 5.27 kPa) when coupled with FSS.

While the expression of late‐stage osteogenic markers, including RUNX2 and OCN, did not show substantial changes, we observed a mild upregulation of OPN. These results can be attributed to the short 7‐day culture duration and are consistent with previous studies that established the primary role of mechanotransduction in guiding the osteogenic commitment of stem cells.^48^, ^81^, ^82^, ^83^ Moreover, they remain consistent with the threshold‐dependent “pressure switch” described by Henstock et al.,^44^ which suggested that higher pressure magnitudes and longer culture times are required to trigger terminal differentiation.

Our findings underscore the intricate nature of mechanotransduction in bone tissue, in which the specific type, magnitude, and combination of mechanical stimuli are paramount in guiding stem cell fate. Furthermore, these results suggest that even subtle mechanical signals generated by low‐magnitude forces can be sufficient to trigger early osteogenic responses. Therefore, precise control and tailoring of the mechanical environment of bone tissue‐like constructs cultured in vitro is a key strategy for advancing bone research and engineering. In this perspective, the tunability of the bioreactor enables stimulation parameters to be tailored to the specific cell type and maturation stage.

Some limitations should be acknowledged in this study. Regarding the intermittent HP unit, pressure values were characterized on specific scaffold types as a function of *T* and DC, since the unit does not operate under closed‐loop pressure control. Although the use of different scaffold types would require a new HP characterization, the identified ranges of *T* and DC are sufficiently broad to enable the delivery of physiologically relevant stimulation across a wide variety of scaffolds. With respect to FSS estimation, a permeability‐based analytical model was adopted, providing scaffold‐scale values while neglecting the contribution of cells and the potential development of extracellular matrix within the construct. Although local FSS may vary spatially within the pore network and temporally during culture, this approach is appropriate for the aim of the study, which focuses on tissue‐scale effects of mechanical cues rather than on a detailed characterization of the FSS distribution within the constructs. Finally, biological tests were conducted over a relatively short culture period (7 days). Nevertheless, this duration was sufficient to capture measurable cellular responses induced by mechanical stimulation and indicative of early osteogenic commitment in BM‐MSCs. Accordingly, pressure values within the physiological range associated with daily activities were selected to provide a favorable mechanical environment for early osteogenic processes while avoiding excessive stimulation. Later stages of differentiation were not investigated, as they were beyond the scope of the present work. In the future, inspired by the work of Henstock et al.,^44^ additional mechanical stimulation protocols could be designed to further expand the scope of the investigations, by increasing the applied intermittent pressure magnitude and by including dynamic adjustment of physical stimulation over time or selection of sex‐specific biomechanical parameters.^84^ Regarding additional physical stimulations, in our previous studies the perfusion bioreactor was combined with a pulsed electro‐magnetic field (PEMF) stimulator (IGEA Clinical Biophysics, Italy) to investigate the combined effects of FSS and PEMF stimulations on bone tissue models.^24^, ^25^ In the perspective of exploring multi‐physical stimulation strategies in bone biology, the proposed FSS & intermittent HP bioreactor could also be integrated with PEMF stimulation, enabling the concurrent application of three distinct physical cues (i.e., FSS, intermittent HP, and PEMF stimulation) to 3D bone tissue models.^85^, ^86^

Finally, the modularity of the developed bioreactor makes it a highly versatile platform, in which each stimulation modality can be applied independently or in combination. This flexibility offers the possibility to design a wide range of experimental conditions aimed at investigating diverse biological scenarios. In future studies, we will explore the simultaneous application of the three physical stimuli, coupled with high‐throughput analyses, to gain deeper insight into the mechanobiological pathways underlying bone regeneration driven by biophysical stimulation.

## 5 CONCLUSIONS

This study presents a bioreactor capable, for the first time, of concurrently delivering controlled and tunable FSS and intermittent HP to 3D bone tissue models. The novelty of the platform lies not only in the combination of physiologically relevant mechanical cues but also in their tunability, which enables designing tailored stimulation protocols to address specific mechanobiological questions.

Comprehensive mechanical characterization demonstrated the versatility of the bioreactor to reproducibly deliver biomimetic stimulation conditions across different 3D bone tissue models. Biological experiments showed that 1 week of combined FSS and intermittent HP stimulation, within physiological ranges associated with daily activities, elicited early osteogenic differentiation. These results support the use of the proposed bioreactor as a robust and versatile platform for in vitro studies of bone mechanobiology.

Finally, the modular architecture of the system allows for integration with additional physical stimuli, such as PEMF, and for the design of more complex multiphysical stimulation strategies.

In the future, coupling the FSS and intermittent HP bioreactor with high‐throughput analyses may provide deeper insight into the molecular mechanobiological mechanisms governing bone regeneration and enable the identification of stimulation regimes tailored to specific osteogenic responses.

## AUTHOR CONTRIBUTIONS

**Stefano Gabetti:** Methodology; software; investigation; formal analysis; visualization; writing – original draft. **Farah Daou:** Methodology; investigation; formal analysis; visualization; writing – original draft. **Beatrice Masante:** Investigation; formal analysis. **Ranveer Kaur:** Investigation; formal analysis. **Simone Israel:** Investigation; formal analysis. **Shahd Alahmad:** Investigation; formal analysis. **Elisa Scatena:** Investigation; resources; writing – original draft. **Carlotta Achille:** Resources. **Raffaella Pecci:** Investigation; resources. **Costantino Del Gaudio Gaudio:** Resources. **Martina Birocco:** Investigation; formal analysis. **Andrea Cochis:** Conceptualization; methodology; supervision; funding acquisition; writing – review and editing. **Lia Rimondini:** Conceptualization; supervision; funding acquisition; project administration; writing – review and editing. **Cristina Bignardi:** Conceptualization; supervision; funding acquisition; project administration; writing – review and editing. **Diana Massai:** Conceptualization; methodology; supervision; funding acquisition; project administration; visualization; writing – review and editing.

## FUNDING INFORMATION

This work was partially supported by the BIGMECH project funded by the Ministero dell'Università e della Ricerca within the PRIN 2022 program (D.D.104‐02/02/2022). This manuscript reflects only the authors' views and opinions, and the Ministry cannot be considered responsible for them.

## CONFLICT OF INTEREST STATEMENT

The authors declare that they have no conflict of interest.

## Supporting information


**Figure S1.** Preview printing of biomimetic scaffold models. (A) PLA600 scaffold; (B) P3S3 scaffold.
**Figure S2**. Pressure measurement using an in‐line physiological relative pressure sensor (SP844, HJK Sensoren, Germany) positioned between the CC and the pinch valve.
**Figure S3**. Cell viability of bone marrow‐derived mesenchymal stem cells (BM‐MSCs) cultured within PLA600 scaffolds for 7 days under static culture (S), direct perfusion (DP), and direct perfusion combined with intermittent pressure simulating daily activity (DP + IP). Live/dead assay imaging reveals the coexistence of dense cell sheets and individual adherent cells exhibiting a characteristic fibroblast‐like morphology.
**Figure S4**. Gene expression analysis of key chondrogenic markers (SOX9 and COL2A1) and proliferation marker (Ki‐67) in bone marrow‐derived mesenchymal stem cells (BM‐MSCs) cultured within PLA600 scaffolds for 7 days. The DP + IP group was compared to the DP group using the 2^−ΔΔ*Cq*^ method. Dots represent the relative quantification of 3 biological replicates, and error bars indicate the mean and the standard error of the mean (s.e.m.).

## Data Availability

The data that support the findings of this study are available from the corresponding author upon reasonable request.
