# Supplementary material for: Parallelized multi‐stimuli bioreactor for combined, physiologically relevant fluid shear stress and intermittent hydrostatic pressure in 3D bone tissue models
Source: Bioeng Transl Med. 2026 Oct 28:e70184. Online ahead of print. doi: 10.1002/btm2.70184 (PMC13714730; doi:10.1002/btm2.70184)
Supplement: Supplementary file 1 [file BTM2-9999-e70184-s001.docx]

***Supplementary material***

**Parallelized multi-stimuli bioreactor for combined, physiologically relevant fluid shear stress and intermittent hydrostatic pressure in 3D bone tissue models**

Stefano Gabetti^1,2*^, Farah Daou^3*^, Beatrice Masante^1,2^, Ranveer Kaur^3^, Simone Israel^1,2^, Shahd Alahmad^3^, Elisa Scatena^4,5^, Carlotta Achille^4^, Raffaella Pecci^6^, Costantino Del Gaudio^7^, Martina Birocco^1,8^, Andrea Cochis^2,3^, Lia Rimondini^2,3^, Cristina Bignardi^1,2^, Diana Massai^1,2^

1. Department of Mechanical and Aerospace Engineering and PolitoBIOMed Lab, Politecnico di Torino, Turin, Italy.
2. Centro 3R, Interuniversity Center for the Promotion of the 3Rs Principles in Teaching and Research, Turin, Italy
3. Dept. of Health Sciences, Center for Translational Research on Autoimmune and Allergic Diseases (CAAD), Università del Piemonte Orientale (UPO), Novara, Italy
4. E. Amaldi Foundation, Rome, Italy,
5. Hypatia Research Consortium, Grottaferrata, Rome, Italy
6. National Center for Artificial Intelligence and Innovative Technologies for Health, Istituto Superiore di Sanità, Rome, Italy
7. Italian Space Agency, Rome, Italy
8. Laboratory for High Performance Ceramics, Empa - Swiss Federal Laboratories for Materials Science and Technology, Dübendorf, Switzerland

* Stefano Gabetti and Farah Daou have contributed equally to this work

**Corresponding author:**

Diana Massai, [diana.massai@polito.it](mailto:diana.massai@polito.it)

Department of Mechanical and Aerospace Engineering and PolitoBIOMed Lab, Politecnico di Torino, Corso Duca Degli Abruzzi, 24, 10129 Turin, Italy

**Biomimetic scaffolds design and manufacturing**

Biomimetic scaffolds were designed according to a previously reported procedure [51]. Firstly, a random 3D distribution of spheres, as virtual porogens, was created by means of a custom-made script. Then, the 3D stochastic arrangement of spheres was subtracted from a cylindrical solid volume (10 mm diameter, 5 mm height), thereby generating a porous architecture. The resulting datasets were exported in STL format and imported in IdeaMaker v3.6.1 software (Raise 3D Inc., USA) for slicing and driving the Raise 3D N2 printer (Raise 3D Inc., USA), see Figure S1. A commercial PLA filament (FILOALFA, Italy) was extruded at 205 °C through a 0.4 mm diameter nozzle, the build platform temperature was set at 60 °C. The manufactured biomimetic scaffolds resulted in a cylindrical shape, with dimensions: diameter = 10 mm; height = 5 mm.

**Figure S1** Preview printing of biomimetic scaffold models. A. PLA600 scaffold; B. P3S3 scaffold.

51. Pecci R, Baiguera S, Ioppolo P, et al (2020) 3D printed scaffolds with random microarchitecture for bone tissue engineering applications: Manufacturing and characterization. J Mech Behav Biomed Mater 103:103583. https://doi.org/10.1016/j.jmbbm.2019.103583

**Bioreactor testing and characterization**

**Intermittent HP**

**Figure S2** Pressure measurement using an in-line physiological relative pressure sensor (SP844, HJK Sensoren, Germany) positioned between the CC and the pinch valve.

**Protocol for scaffold permeability measurement**

Scaffold permeability was assessed using a custom permeability test bench (PTB) and an established protocol, previously developed and described [57].

Each scaffold sample was first measured with a caliper to record its dimensions and was then inserted press-fit into a tailored flexible gasket to ensure a watertight seal. The sample-gasket assembly was mounted into the two-part permeability chamber (PC) housing, which was then closed with screws and connected to the hydraulic circuit. The circuit was filled with 500 mL of demineralized water at room temperature, and the pump was activated to remove air and wet the sample. The system was operated overnight to ensure full saturation. Before each test, the pump was stopped and pressure sensors (upstream and downstream of the sample) were zeroed.

To maintain laminar flow within the sample, it was verified that the interstitial Reynolds number (*Re*) satisfied the laminarity condition for fluid inside complex porous media:

$$Re=\frac{\rho vD}{\mu}<1$$

where *v* is the fluid linear velocity, *D* is the mean pore diameter of the sample, ρ is the density of the testing fluid (ρ = 998 kg/ m^3^), and *μ* is its dynamic viscosity (*μ* = 1.002 × 10^–3^ Pa∙s at 20 °C). Thus, considering the geometrical features of the scaffolds, a flow rate *Q* = 5 mL/min was selected, as the Reynolds number resulted: *Re_PLA600_* = 0.671 and *Re_P3S3_* = 0.930.

Experiments were conducted on 4 samples, with 3 repetitions per sample. During each test, five pressure measurements were taken: one over a 1-hour period, followed by four 15-minute intervals. Additional measurements were performed without a sample to isolate the pressure drop due solely to the sample. Flow rate validation was conducted post-test by measuring the volume of water over a 2-minute interval using a scale.

Intrinsic permeability (*k*) was calculated using Darcy’s law:

$$k=\mu\frac{Q}{\Delta p}\frac{L}{A}$$

where *Δp* is the measured pressure drop across the scaffold, *L* is the thickness of the scaffold and *A* is the cross-sectional area of the scaffold.

57. Gabetti S, Masante B, Schiavi A, et al (2024) Adaptable test bench for ASTM-compliant permeability measurement of porous scaffolds for tissue engineering. Sci Rep 14:1722. https://doi.org/10.1038/s41598-024-52159-4

**Biological tests**

**Cell viability, infiltration, and distribution assessments**

**Figure S3.** Cell viability of bone marrow-derived mesenchymal stem cells (BM-MSCs) cultured within PLA600 scaffolds for 7 days under static culture (S), direct perfusion (DP), and direct perfusion combined with intermittent pressure simulating daily activity (DP+IP). Live/dead assay imaging reveals the coexistence of dense cell sheets and individual adherent cells exhibiting a characteristic fibroblast-like morphology.

**Gene expression analysis**

**Figure S4.** Gene expression analysis of key chondrogenic markers (SOX9 and COL2A1) and proliferation marker (Ki-67) in bone marrow-derived mesenchymal stem cells (BM-MSCs) cultured within PLA600 scaffolds for 7 days. The DP+IP group was compared to the DP group using the 2^−ΔΔCq^ method. Dots represent the relative quantification of 3 biological replicates, and error bars indicate the mean and the standard error of the mean (s.e.m.).
